# Erythrocytes Induce Endothelial Injury in Type 2 Diabetes Through Alteration of Vascular Purinergic Signaling

**DOI:** 10.3389/fphar.2020.603226

**Published:** 2020-11-30

**Authors:** Ali Mahdi, Yahor Tratsiakovich, John Tengbom, Tong Jiao, Lara Garib, Michael Alvarsson, Jiangning Yang, John Pernow, Zhichao Zhou

**Affiliations:** ^1^ Division of Cardiology, Department of Medicine, Karolinska Institutet, Stockholm, Sweden; ^2^ Division of Endocrinology and Diabetology, Department of Molecular Medicine and Surgery, Karolinska Institutet, Stockholm, Sweden; ^3^ Department of Cardiology, Karolinska University Hospital, Stockholm, Sweden

**Keywords:** erythrocyte, endothelial dysfunction, diabetes, purinergic receptor, adenosine triphosphate, adenosine

## Abstract

It is well established that altered purinergic signaling contributes to vascular dysfunction in type 2 diabetes (T2D). Red blood cells (RBCs) serve as an important pool for circulating ATP and the release of ATP from RBCs in response to physiological stimuli is impaired in T2D. We recently demonstrated that RBCs from patients with T2D (T2D RBC) serve as key mediators of endothelial dysfunction. However, it remains unknown whether altered vascular purinergic signaling is involved in the endothelial dysfunction induced by dysfunctional RBCs in T2D. Here, we evaluated acetylcholine-induced endothelium-dependent relaxation (EDR) of isolated rat aortas after 18 h *ex vivo* co-incubation with human RBCs, and aortas of healthy recipient rats 4 h after *in vivo* transfusion with RBCs from T2D Goto-Kakizaki (GK) rats. Purinergic receptor (PR) antagonists were applied in isolated aortas to study the involvement of PRs. EDR was impaired in aortas incubated with T2D RBC but not with RBCs from healthy subjects *ex vivo,* and in aortas of healthy rats after transfusion with GK RBCs *in vivo*. The impairment in EDR by T2D RBC was attenuated by non-selective P1R and P2R antagonism, and specific A1R, P2X_7_R but not P2Y_6_R antagonism. Transfusion with GK RBCs *in vivo* impaired EDR in aortas of recipient rats, an effect that was attenuated by A1R, P2X_7_R but not P2Y_6_R antagonism. In conclusion, RBCs induce endothelial dysfunction in T2D via vascular A1R and P2X_7_R but not P2Y_6_R. Targeting vascular purinergic singling may serve as a potential therapy to prevent endothelial dysfunction induced by RBCs in T2D.

## Introduction

Type 2 diabetes (T2D) is an important risk factor for the development of cardiovascular disease including ischemic heart disease and myocardial infarction (Paneni et al., 2013). Both microvascular and macrovasular complications significantly contribute to the increase in mortality and morbidity in the large population with T2D (Paneni et al., 2013). Endothelial dysfunction plays a pivotal role in the etiology of T2D-induced vascular complications. This is characterized by an imbalance between endothelium-derived vasodilators such as nitric oxide (NO) and adenosine triphosphate (ATP), and vasoconstrictors such as reactive oxygen species (ROS) and ATP including its down-stream purinergic signaling (Gimbrone and Garcia-Cardena, 2016; Mahdi et al., 2018). The underlying disease mechanisms for the development of endothelial dysfunction in T2D are complex and not fully understood. There is a clinical need for improved understanding of these disease mechanisms in order to develop new therapeutic strategies for the treatment of vascular complications.

Red blood cells (RBCs) play a crucial role in cardiovascular homeostasis due to their primary function as gas carrier. We have recently unveiled that RBCs not only exert physiological functions but also act as important trigger and mediator for endothelial dysfunction in T2D (Pernow et al., 2019). Thus, RBCs from patients with T2D caused endothelial dysfunction in healthy arteries (Zhou et al., 2018). Intriguingly, the detrimental effect of RBCs from T2D patients on endothelial function remained following improved glycemic control indicating that the mechanism behind this effect is not explained by hyperglycemia only (Mahdi et al., 2019). The mechanisms underlying this novel function of RBCs for the development of endothelial dysfunction in T2D remain incompletely understood and need further investigations.

RBCs serve as an ATP pool in the circulation. RBCs release ATP in response to low oxygen tension and/or mechanical deformation (Sprague and Ellsworth, 2012). Once released, ATP plays a fundamental role in regulating blood flow and tissue perfusion via activation of purinergic receptors (PRs) in the vasculature (Sprague and Ellsworth, 2012; Zhou et al., 2020). PRs have been classified into two subtypes: P1Rs and P2Rs. Four subtypes of P1Rs (also termed adenosine receptors) have been identified, namely A1R, A_2A_R, A_2B_R and A3R. Seven P2XR and eight P2YR have been cloned to date (Burnstock and Novak, 2013; Wernly and Zhou, 2020; Zhou et al., 2020). Interestingly, the release of ATP from RBCs is decreased in patients with T2D, and this impairment in ATP release is associated with attenuated vasodilation in arteries incubated with RBCs from patients with T2D (Sprague et al., 2011). Moreover, we and others previously demonstrated that purinergic signaling is altered in T2D contributing to vascular dysfunction (Burnstock and Novak, 2013; Zhou et al., 2017; Mahdi et al., 2018; Zhou et al., 2020). However, whether RBCs from patients with T2D alter vascular purinergic signaling accounting for endothelial dysfunction is largely unknown.

Consequently, we tested the hypothesis that RBCs induce endothelial dysfunction in T2D through alteration of vascular purinergic signaling. We performed experiments in a well-established *ex vivo* co-incubation system using RBCs isolated from patients with T2D as well as healthy subjects for assessment of endothelial function (Zhou et al., 2018). We also investigated the detrimental effect of RBCs from patients with T2D in an *in vivo* rat RBC transfusion model. By using both the non-selective and the selective receptor antagonists, we demonstrate a pivotal role of P1R and P2R in the development of endothelial dysfunction induced by RBCs in patients with T2D.

## Materials and Methods

### Study Subjects

Seventeen patients with T2D were recruited from the Department of Endocrinology, Karolinska University Hospital. T2D was defined according the World Health Organization criteria. Fifteen age-matched healthy controls that were free of medication, and had no medical history of any cardiovascular disease were recruited. Subject characteristics are summarized in Table 1. Following an overnight fasting period, whole blood was collected in heparinized tubes with subsequent isolation of RBCs by immediate centrifugation at +4°C and 1000 g for 10 min followed by three washing cycles with Krebs-Henseleit (KH) buffer (Zhou et al., 2018). This procedure could successfully remove >99% white blood cells and ≥98% platelets (Yang et al., 2013). All samples with hemolysis were excluded from the study. The procedure was conducted according the principles outlined by the declaration of Helsinki and was approved by the regional ethical review board in Stockholm. All subjects were informed of the purpose and gave their oral and written informed consent.

**TABLE 1:** Subject characteristics.

Variables:	Healthy subjects n = 15	Type 2 diabetes n = 17
Age	61 ± 6	63 ± 12
No. of males	7	15
BMI, kg/m^2^	23 ± 2	32 ± 5***
BP, mmHg		
Systolic	130 ± 19	137 ± 13
Diastolic	81 ± 7	83 ± 9^a^
Fasting glucose, mM	5.6 ± 0.4	11.2 ± 3.8***
No. of smokers	0	1
HbA1c, mmol/mol	35 ± 2	69 ± 23***^a^
Hemoglobin, g/L	142 ± 9	146 ± 18
Creatinine, mmol/L	78 ± 14	94 ± 36^a^
Triglycerides, mmol/L	1.4 ± 1.1	2.1 ± 1.1*^a^
Total cholesterol, mmol/L	5.5 ± 1.0	4.4 ± 1.4*
HDL, mmol/L	1.7 ± 0.3	1.1 ± 0.2***
LDL, mmol/L	3.4 ± 0.9	2.3 ± 1.0**
Comorbidity (n):		
CAD	0	2
Retinopathy	0	2
Neuropathy	0	3
Nephropathy	0	1
Peripheral vascular disease	0	1
Medication (n):		
ACEi/ARB	0	8
Aspirin	0	6
Lipid lowering	0	13
β-blockers	0	2
Calcium channel i	0	6
Insulin	0	10
Metformin	0	16
GLP1 analogue	0	2
DDP-4i	0	2
SU	0	3
SGLT2i	0	3

ACEi = angiotensin-converting enzyme inhibitor; ARB = angiotensin receptor blocker; BMI = body mass index; BP = blood pressure; CAD: coronary artery disease; DPP-4i = dipeptidyl peptidase-4 inhibitor; GLP-1 = glucagon like peptide-1; HbA1c = glycated hemoglobin; HDL = high-density lipoprotein; LDL = low-density lipoprotein; SGLT2i = sodium-glucose co-transporter inhibitor; SU = sulfonylurea. Values are mean ± SD; *p <0.05, **p <0.01, ***p <0.001 vs. healthy subjects.

a Analyzed by Mann-Whitney; the remaining parameters were analyzed by unpaired *t*-test.

### Animals

Male Wistar rats aged 10-20 weeks (Charles-River, Sulzfeld, Germany) were anesthetized with pentobarbital (50 mg/kg, i.p.) followed by thoracotomy and isolation of thoracic aortic segments. Male non-obese T2D Goto-Kakizaki (GK) rats were bred at the animal facility of the Karolinska University Hospital and used at age of 15-18 weeks for the RBC transfusion experiments. Animal care and all protocols were approved by the regional ethical committee and conformed to the Guide for Care and Use of Laboratory Animals published by the US National Institutes of Health (NIH publication No. 85-23, revised 1996).

### RBC-Tissue Co-Incubation and Myograph Studies

Washed RBCs from patients with T2D (T2D RBC) and healthy subjects (H RBC) were diluted to a hematocrit of ∼45% with KH buffer and co-incubated with rat aortas in cell culture incubator at 37°C with 95% O_2_ and 5% CO_2_ for 18 h (Zhou et al., 2018). Following the incubation, vessels were carefully washed and mounted the wire myographs (Danish Myo Technology, Denmark). Contractility of vessels were examined with KCl (50 and 100 mM). Endothelium-dependent relaxation (EDR) was determined by application of cumulatively increasing concentrations (10^−9^-10^−5^ M) of acetylcholine (ACh) to aortic segments preconstricted with 9,11-Dideoxy-9α,11α-methanoepoxy prostaglandin 2α (U46619, 30 nM). A previous study demonstrated that endothelium-independent relaxations are unaffected by T2D RBC (Zhou et al., 2018). We have previously demonstrated that the free hemoglobin to RBC hemoglobin ratio after 18h incubation is negligible and does not differ between RBCs from healthy and T2D patients (Zhou et al., 2018). For determination of vascular PR involvement, the non-selective P1R antagonist 8PT (10 µM), the non-selective P2R antagonist PPADS (10 µM), as well as the cardiovascular relevant and specific PR antagonists: the A1R antagonist DPCPX (10 nM), the P2X_7_R antagonist A438079 (10 µM), and the P2Y_6_R antagonist MRS2578 (10 µM) were added in the organ bath for 30 min where the vessels were present following the 18h incubation with RBCs. EDR was then determined in U46619-preconstricted vessels (Mahdi et al., 2018).

### Rat RBC Transfusion

Recipient Wistar rats were anesthetized with pentobarbital 50 mg/kg, i.p. and placed on a heated pad to maintain body temperature at 37.5–38.5°C. The left jugular vein was cannulated with a PE-10 catheter for administration of pentobarbital during the experiment. The right carotid artery was cannulated with a PE-50 catheter filled with heparinized saline. The animals were tracheotomized, intubated and ventilated with room air (56 strokes/min, 9 ml/kg tidal volume). After 15 min stabilization 1 ml washed RBCs from donor rats (GK and Wistar) were resuspended in 1 ml PBS, kept at 37°C in a syringe and connected to the three way stop cock that was attached to the carotid catheter. Another syringe was used to remove 2 ml blood from the recipient rat after which 2 ml of RBCs from the donor rats were immediately infused into the carotid artery. The animal was kept anaesthetized for 4 h after which the animal was sacrificed and the aorta was harvested for vessel reactivity studies in the absence and presence of DPCPX (10 nM), A438079 (10 µM) and MRS2578 (10 µM). All recipient rats and donor rats were age matched. The information of animals used for the RBC transfusion is listed in Supplementary Table S1. RBC transfusion in rodents has been carried out with negligible side effects (Huang et al., 2017), which is supported by our preliminary observations that the level of plasma free hemoglobin in relation to whole blood free hemoglobin was <1% in recipients following the RBC transfusion.

### Statistical Analysis

Data are presented as means ± SD. Vascular relaxation to ACh was expressed as percentage of contraction to U46619 (Zhou et al., 2018; Mahdi et al., 2019). Differences in concentration-dependent relaxations induced by ACh were analyzed using two-way ANOVA followed by Bonferroni’s test when appropriate. Differences between two groups were performed using unpaired two-tailed *t*-test or non-parametric Mann-Whitney test when appropriate, while differences among multiple groups were analyzed using one-way ANOVA followed by Bonferroni’s test. Normal distribution of data was tested using d’Agostino-Pearsons normality test. The number of experimental observations (n) refers to the number of animals and RBC donors included. We used one RBC donor for one rat donor. When vessel segments were incubated with buffer only, n refers to the numbers of animals. The statistical analysis was calculated based on the n. All analysis was calculated using GraphPad Prism (V6.05). Statistical significance was accepted when *p* < 0.05.

## Results

### Subject Characteristics

Fasting blood glucose, glycated hemoglobin (HbA1c) and body mass index (BMI) were significantly higher in patients with T2D compared to healthy controls (Table 1). Blood lipids except for triglycerides were significantly lower in patients with T2D (Table 1).

### RBCs Induce Endothelial Dysfunction in T2D *Ex Vivo* and *In Vivo*

**FIGURE 1:**
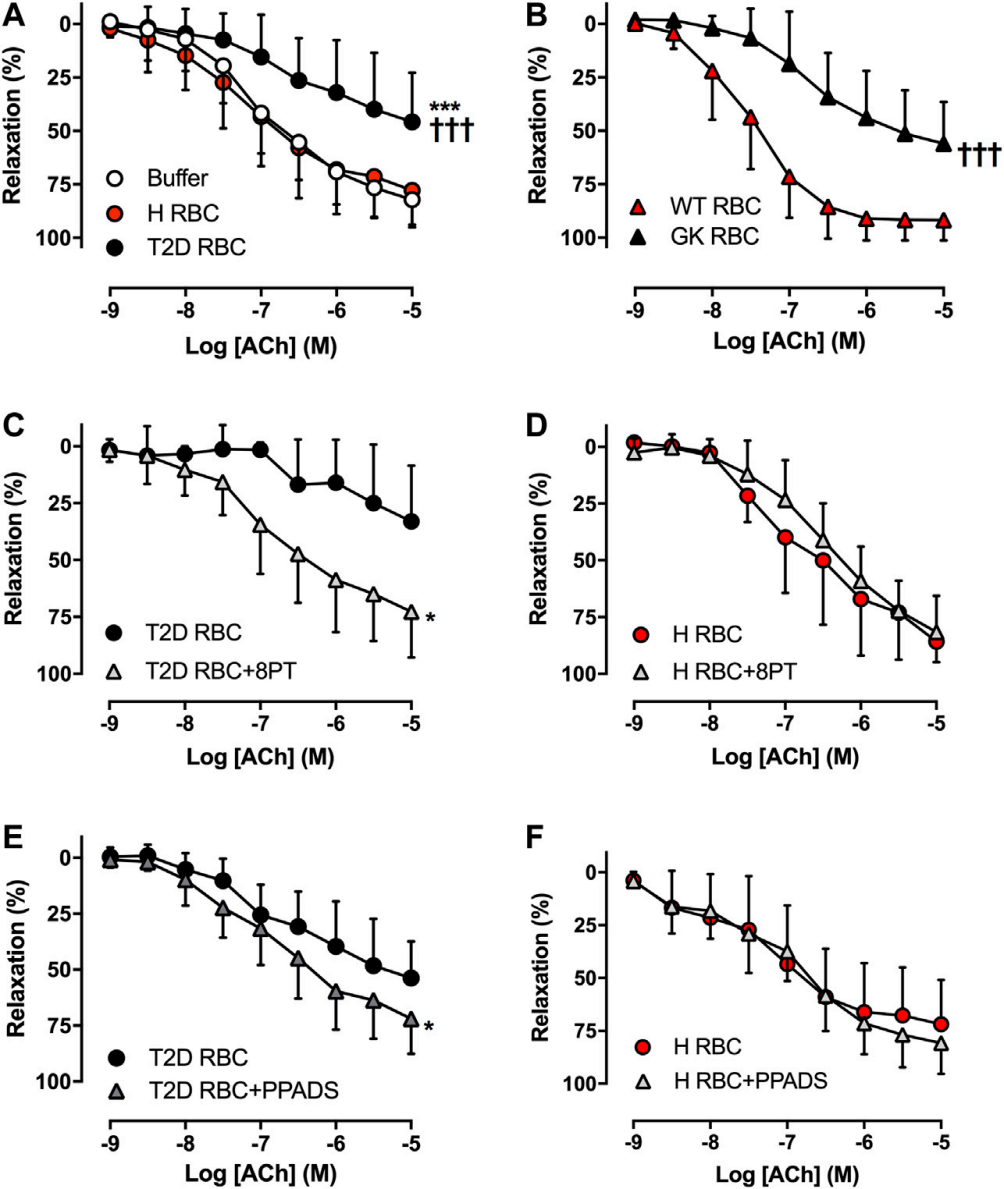
Effect of red blood cells (RBCs) on endothelial function. Effect of RBCs from type 2 diabetes patients (T2D RBC), healthy subjects (H RBC) or buffer only on endothelium-dependent relaxation (EDR) in rat aortas induced by acetylcholine (ACh) (**A**: n = 15-17). EDR in aortas of healthy recipient rats transfused with RBCs from GK (GK RBC) or Wistar rats (WT RBC) (**B**: GK RBC n = 12, WT RBC n = 6). Effect of the non-selective P1R antagonist 8PT and the non-selective P2R antagonist PPADS on EDR in rat aortas incubated with T2D RBC (**C**: n = 8, **E**: n = 5) or H RBC (**D**: n = 4, **F**: n = 6). Values are mean ± SD. *p < 0.05 effect of antagonist; ***p < 0.01 vs. buffer; †††*p* < 0.001 vs. H RBC or WT RBC.

The U46619- and KCl-induced contraction in aortas incubated with T2D RBC were comparable with that in aortas incubated with H RBC (Supplementary Figure S1). In accordance with our recent findings using the well-established *ex vivo* human RBC-vessel co-incubation model (Zhou et al., 2018; Mahdi et al., 2019; Mahdi et al., 2020), T2D RBC but not H RBC or buffer induced vascular endothelial dysfunction, which is evident from the significant impairment in EDR in rat aortas incubated with T2D RBC (Figure 1A). Further, we performed rat RBC transfusions (hematocrit ∼45%) to unveil the effect of RBCs on endothelial function *in vivo*. Transfusion of RBC from Wistar to Wistar rats did not induce endothelial dysfunction. By contrast, EDR was significantly impaired in rat aortas of recipient Wistar rats transfused with RBCs from GK rats (GK RBC) (Figure 1B). This observation is in line with our *ex vivo* findings and an important extension of the findings obtained from the *ex vivo* RBC-vessel co-incubation system.

### Involvement of Vascular PRs in Endothelial Dysfunction Induced by RBCs in T2D

**FIGURE 2:**
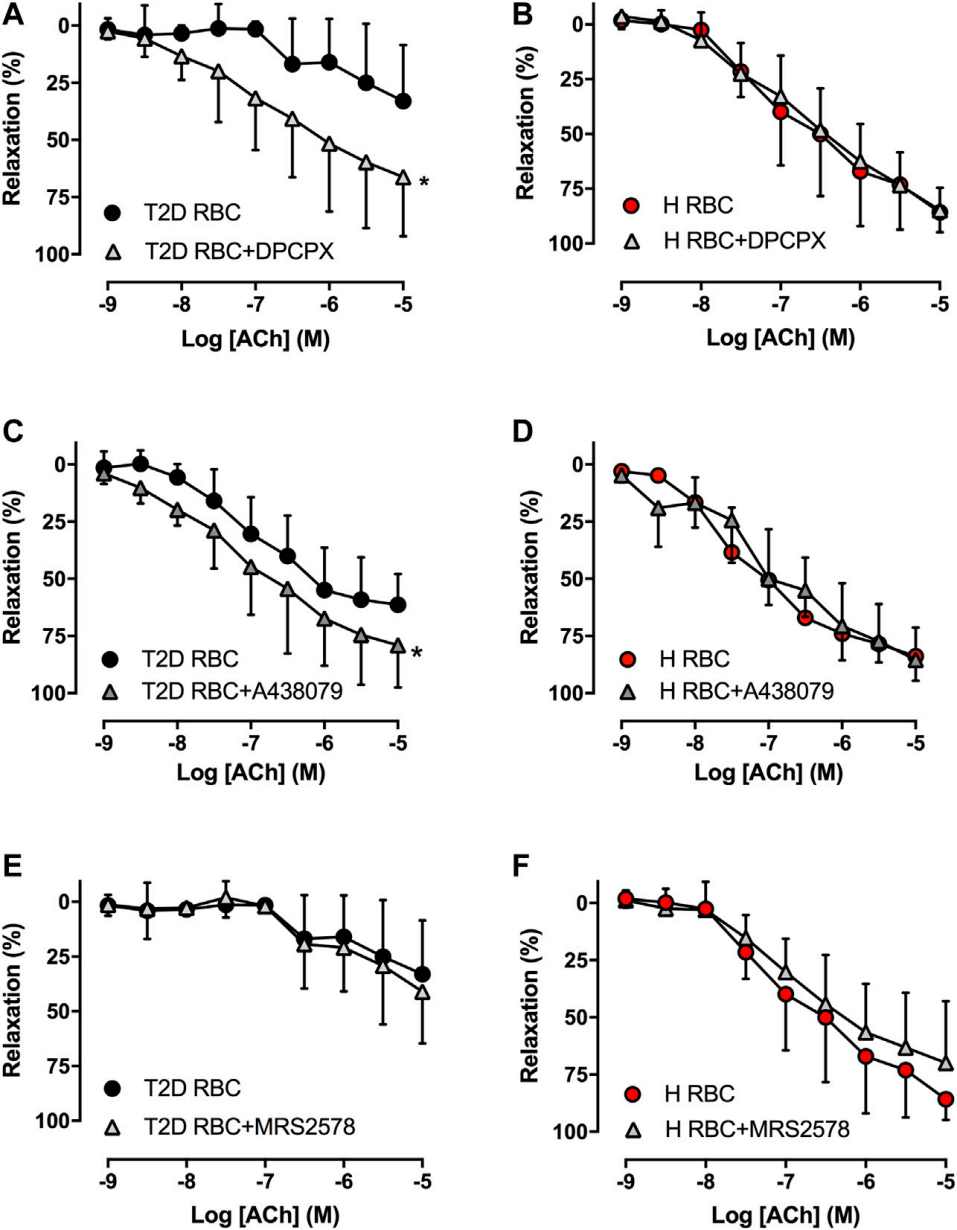
Effect of the A1R antagonist DPCPX, the P2X_7_R antagonist A438079 and the P2Y_6_R antagonist MRS2578 on EDR in rat aortas incubated with T2D RBC (**A**: n = 8, **C**: n = 4, **E**: n = 7-8) or H RBC (**B**: n = 4, **D**: n = 5, **F**: n = 4). Values are mean ± SD. *p < 0.05 effect of antagonist.

**FIGURE 3:**
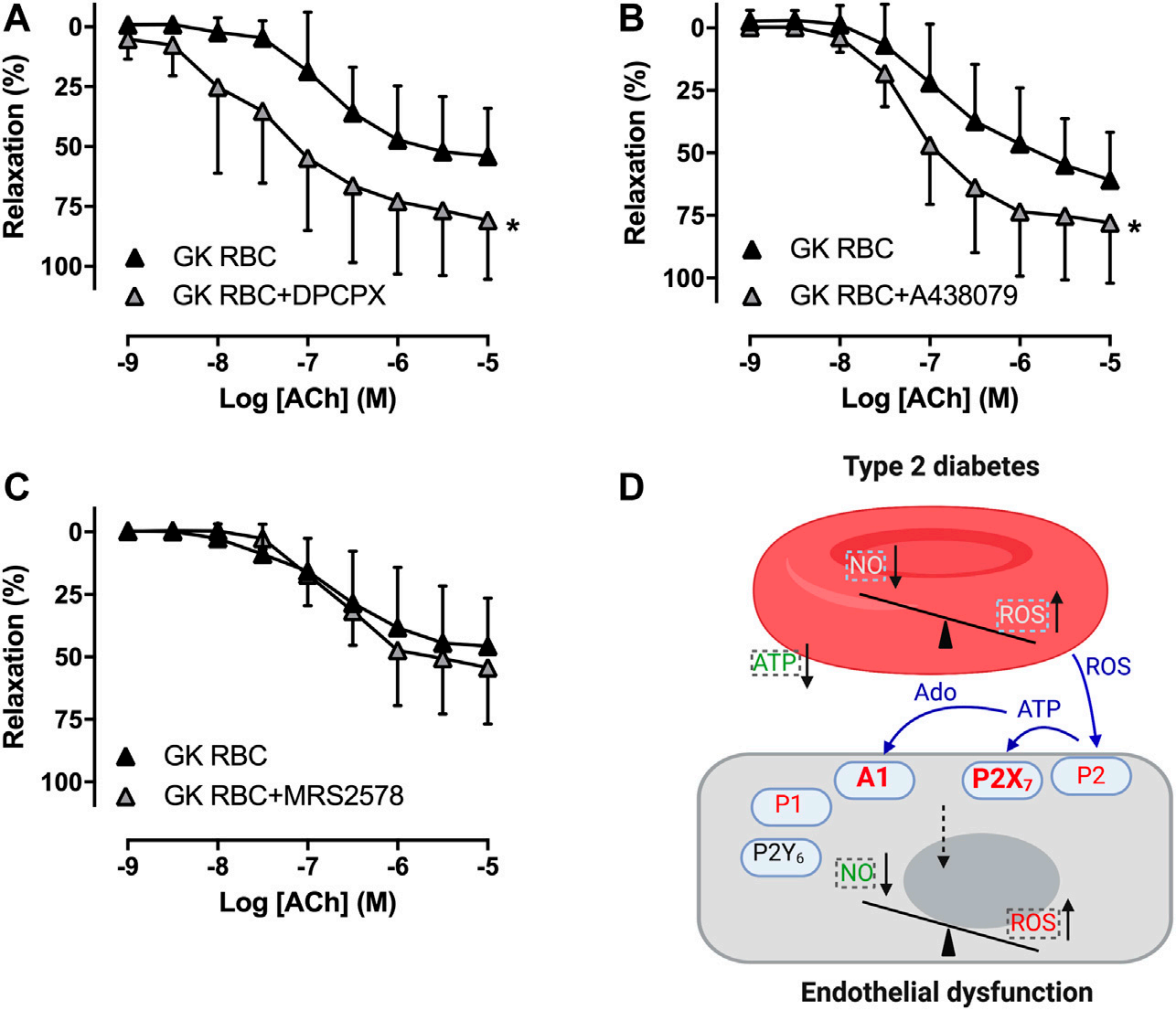
Effect of purinergic receptor inhibition on endothelial function of aortas in recipient rats transfused with rat RBCs. Effect of the A1R antagonist DPCPX, the P2X_7_R antagonist A438079 and the P2Y_6_R antagonist MRS2578 on EDR in aortas of rats transfused with GK RBC (**A**: n = 6, **B**: n = 8, **C**: n = 6). Schematic summary of the present study **(D)**: Dysfunctional RBCs of T2D alter vascular A1R and P2X_7_R but not P2Y_6_R accounting for endothelial dysfunction. Dashed boxes represent earlier findings where there are decreased nitric oxide (NO) bioavailability, increased reactive oxygen species (ROS) formation/release and impaired release of ATP from RBCs of T2D that break the balance between NO bioavailability and ROS in the endothelium (Pernow et al., 2019). Signaling in blue color represents the hypothesized mechanisms that the increased formation of ROS derived from RBCs may stimulate ATP release in other (endothelial) cells than RBCs to activate P2X_7_R in endothelium. Together with the subsequent degradation product adenosine, vasoconstrictor A1R and P2X_7_R are activated in T2D accounting for endothelial dysfunction. Values are mean ± SD. *p < 0.05 effect of antagonist. Ado: adenosine.

To study whether RBCs alter vascular purinergic signaling in T2D accounting for endothelial dysfunction using the well-established *ex vivo* human RBC-vessel co-incubation model, the non-selective P1R (8PT) and P2R antagonists (PPADS) were applied in the organ bath. Both 8PT (Figure 1C) and PPADS (Figure 1E) significantly attenuated endothelial dysfunction induced by T2D RBC, while these antagonists had no effects on EDR in rat aortas incubated with H RBC (Figures 1D,F). To further investigate the specific PRs involved, different antagonists were applied. The A1R antagonist DPCPX, the P2X_7_R antagonist A438079 but not the P2Y_6_R antagonist MRS2578 attenuated endothelial dysfunction induced by T2D RBC (Figures 2A,C,E). None of the three antagonists had any effect on EDR in rat aortas incubated with H RBC (Figures 2B,D,F). Moreover, we performed rat RBC transfusions to study the involvement of PRs in endothelial dysfunction *in vivo*. The A1R antagonist DPCPX, the P2X_7_R antagonist A438079 but not the P2Y_6_R antagonist MRS2578 attenuated endothelial dysfunction in aortas of recipient rats transfused with GK RBCs (Figures 3A–C). Both *ex vivo* and *in vivo* findings indicate that the RBC-induced endothelial dysfunction in T2D is mediated through alteration of purinergic signaling in the vasculature.

## Discussion

The main findings of the present study are that 1) T2D RBC-induced endothelial dysfunction observed in an *ex vivo* model was reproduced *in vivo* using transfusion of RBCs, 2) non-selective antagonism of P1R and P2R and selective antagonism of A1R, P2X_7_R but not P2Y_6_R in the vasculature attenuated endothelial dysfunction induced by T2D RBC, and 3) selective antagonism of A1R, P2X_7_R but not P2Y_6_R attenuated endothelial dysfunction in vessels of recipient rats transfused with RBCs from diabetic rats. This study provides important information regarding the mechanism underlying endothelial dysfunction associated with T2D by demonstrating that RBCs alter vascular purinergic signaling which results in endothelial dysfunction (Figure 3D).

Several lines of evidence have revealed that RBCs contribute to vascular homeostasis and integrity in addition to their function as gas transporters (Kuhn et al., 2017; Pernow et al., 2019). Of further interest is that RBCs undergo functional changes which includes decreased export of NO bioactivity and increased formation of ROS in several pathophysiological conditions including T2D (Kuhn et al., 2017; Yang et al., 2018; Pernow et al., 2019; Sun et al., 2019). Using an *ex vivo* RBC-vessel co-incubation model, we recently demonstrated that RBCs from patients and rats with T2D induce endothelial dysfunction in both human and rat arteries through mechanisms involving decreased NO bioactivity and increased oxidative stress both at the RBC and vascular levels (Zhou et al., 2018; Pernow et al., 2019; Mahdi et al., 2020) (Figure 3D). The detrimental effect of RBCs of T2D is species-independent and available data suggest that it is the T2D *per se* that contributes to the detrimental effect of RBCs rather than co-morbidities, co-medication, or other confounding factors (Zhou et al., 2018). In the present study, we could not only reproduce this altered function of T2D RBC to induce endothelial dysfunction *ex vivo* but also provide important *in vivo* evidence that transfusion of GK RBCs into healthy rats induces endothelial dysfunction. This strongly indicates that RBCs are capable of causing endothelial dysfunction also under *in vivo* conditions, which is an important extension of the results obtained using the static RBC-vessel co-incubation model.

RBCs can release ATP in response to hypoxia or mechanical stimuli in the circulation (Sprague and Ellsworth, 2012). By contrast, existing evidence suggest that ATP release from RBCs is impaired in response to hypoxia in T2D, which affects vasodilation in pressurized arterioles (Sprague et al., 2011). ATP plays a crucial role in the regulation of blood flow and tissue perfusion via activation of PRs (Burnstock and Novak, 2013; Zhou et al., 2020). The well-balanced relation between vasodilator PRs and vasoconstrictor PRs under healthy conditions may switch to an increased activation of vasoconstrictor PRs and decreased activation of vasodilator PRs in T2D leading to vascular dysfunction (Zhou et al., 2020). We hypothesized that T2D RBC affects vascular purinergic signaling accounting for endothelial dysfunction. Accordingly, we observed that not only non-selective antagonism of vascular P1Rs and P2Rs but also the selective antagonism of vascular A1R and P2X_7_R attenuated endothelial dysfunction induced by T2D RBC. Consistent with this is the observation from the RBC transfusion experiments which revealed that both vascular A1R and P2X_7_R are involved in the development of endothelial dysfunction following transfusion of RBCs from GK rats *in vivo*. We have recently demonstrated that several PRs including A1R and P2X_7_R are involved in the development of endothelial dysfunction in GK rats (Mahdi et al., 2018). The involvement of these receptors depends on their alteration in receptor sensitivity rather than expression (Mahdi et al., 2018), suggesting a potential disease mechanism via alteration of post-PR signaling. The present findings suggest that altered RBC function may affect these two receptors and partially contribute to endothelial dysfunction present in GK rats.

Activation of A1R in endothelial cells typically produces vascular contraction through the thromboxane, PKC and ERK/2 pathways (Zhou et al., 2015; Yadav et al., 2016; Zhou et al., 2020). The vasoconstrictor response to A1R stimulation can be increased in disease states driven by endothelial dysfunction (Yadav et al., 2019). The results of the present study suggest that RBCs induces endothelial dysfunction via activation of endothelial vasoconstrictor A1R in T2D. Existing data have revealed that activation of the P2X_7_R plays a crucial role in the development of inflammation and vascular dysfunction in T2D (Wernly and Zhou, 2020; Zhou et al., 2020). Furthermore, a previous study showed that activation of P2X_7_R in endothelial cells under high glucose stimulation resulted in generation of ROS and endothelial dysfunction (Sathanoori et al., 2015). Our data suggest that RBCs activate endothelial P2X_7_R in T2D leading to endothelial dysfunction. However, these results obtained from *in vitro* experiments may not fully represent the *in vivo* situation of T2D as recently exemplified by the unaltered harmful response to RBCs from patients with T2D following improved hyperglycemic control (Mahdi et al., 2019). Acute elevation of glucose *in vitro* can initiate the harmful cascade for ROS production and induction of endothelial dysfunction, whereas a long-term hyperglycemic state in patients results in a “hyperglycemic memory” involving a complex interplay of multiple factors including ROS that may induce irreversible RBC and endothelial dysfunctions (Costantino et al., 2015). In contrast to the involvement of the P2X_7_R, the P2Y_6_R does not appear to be involved in endothelial dysfunction induced by T2D RBC *ex vivo* and GK RBC *in vivo*. Our recent study has shown that P2Y_6_R is involved in endothelial dysfunction in arteries isolated from GK rats (Mahdi et al., 2018). This may suggest that the activation of P2Y_6_R for the induction of endothelial dysfunction in GK rats is likely attributed to signaling initiated from endothelial cells or additional cell types other than RBCs. Moreover, the lack of involvement of the P2Y_6_R is likely due to the vasodilator property of the receptor activation (Zhou et al., 2017; Kobayashi et al., 2018), an effect that has been shown to be enhanced in a diabetic and obese Otsuka Long-Evans Tokushima Fatty rats (Kobayashi et al., 2018).

The ATP release from RBCs is impaired in T2D resulting in vascular dysfunction, suggesting an alteration of purinergic signaling between RBCs and the vasculature (Sprague and Ellsworth, 2012). However, how RBCs from T2D patients dysregulate vascular A1R and P2X_7_R are not readily known. Increased formation of ROS in T2D leads to high levels of ATP in the circulation and activation of P2R including P2X_7_R resulting in cell dysfunction and death (Rodrigues et al., 2018). We recently demonstrated that RBCs induce endothelial dysfunction in T2D likely via mechanisms involving ROS as a signal between RBCs and the endothelium (Zhou et al., 2018). The increased formation of ROS derived from RBCs may stimulate ATP release in other cells than RBCs to activate P2X_7_R in endothelium. Together with the subsequent degradation product adenosine, vasoconstrictor A1R and P2X_7_R are activated in T2D accounting for endothelial dysfunction (Figure 3D). Possible explanations of these observations may point to an imbalance between decreased RBC-derived ATP, which subsequently results in less activation of vasodilator purinergic receptors on the one hand, and increase in RBC-derived ROS and subsequent ATP release in other cell-mediated vasoconstrictor purinergic receptors on the other hand. Collectively, the net effect determines vascular function in T2D. However, future studies are needed to elucidate the exact signaling pathways transmitted from RBCs that activates vascular purinergic signaling accounting for endothelial dysfunction in T2D.

In conclusion, our study provides important information by demonstrating that RBCs induce endothelial injury in T2D through alteration of vascular purinergic signaling. Targeting vascular purinergic signaling may provide a novel therapy for the treatment of endothelial dysfunction among patients with T2D.

## Data Availability Statement

The original contributions presented in the study are included in the article/Supplementary Material, further inquiries can be directed to the corresponding author.

## Ethics Statement

The studies involving human participants were reviewed and approved by the regional ethical review board in Stockholm. The patients/participants provided their written informed consent to participate in this study. The animal study was reviewed and approved by The regional ethical committee.

## Author Contributions

ZZ conceived and designed the study; AM, YT, JT, TJ, LG, BW, and ZZ performed and collected research data; AM and ZZ analyzed research data and performed statistical analyses; AM, YT, JT, TJ, LG, BW, MA, JY, JP, and ZZ contributed to discussion; ZZ wrote the manuscript; AM, YT, JP, and ZZ edited the manuscript, and all authors reviewed the final version of the manuscript.

## Conflict of Interest

The authors declare that the research was conducted in the absence of any commercial or financial relationships that could be construed as a potential conflict of interest.
