# Supplementary material for: Erythrocytes Induce Endothelial Injury in Type 2 Diabetes Through Alteration of Vascular Purinergic Signaling
Source: Front Pharmacol. 2020 Nov 30;11:603226. doi: 10.3389/fphar.2020.603226 (PMC7774325; doi:10.3389/fphar.2020.603226)
Supplement: Supplementary file 1 [file datasheet1.pdf]

Supplementary materials

Table 1. Characteristics of rats for RBC transfusion

	**Wistar to Wistar**		**GK to Wistar**	
	Wistar donor	Wistar recipient	GK donor	Wistar recipient
n	6	6	12	12
BW	515±55	499±58	334±36***	515±85
B-glucose	3.8±0.3	3.7±0.8	9.1±2.0***	4.1±0.7

*BW: body weight; GK: Goto-Kakizaki. Values are mean±SD; ***P<0.001 vs. values in the rest of columns by one-way ANOVA followed by Bonferroni's test.*

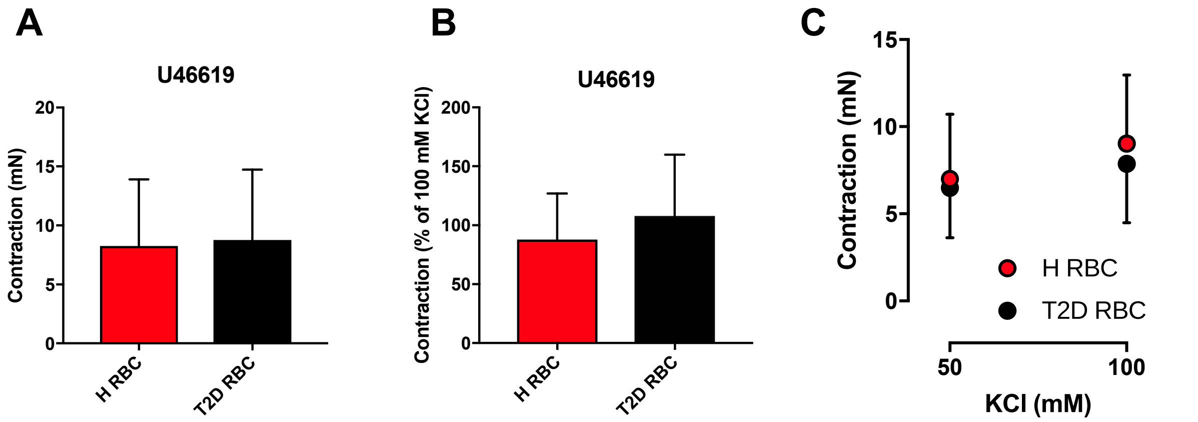

Figure 1. Effects of U46619 and KCl on vascular contraction. (A and B) Effects of U46619 on vascular contraction in aortas following incubation with RBCs from healthy subjects (H RBC, n=15) and type 2 diabetic patiens (T2D RBC, n=17). (C) Effects of different concentrations of KCl on vascular contraction in aortas following incubation with H RBC (n=15) and T2D RBC (n=17). Values are mean ± SD.
